# Quali–Quantitative Fingerprinting of the Fruit Extract of *Uapaca bojeri* Bail. (*Euphorbiaceae*) and Its Antioxidant, Analgesic, Anti-Inflammatory, and Antihyperglycemic Effects: An Example of Biodiversity Conservation and Sustainable Use of Natural Resources in Madagascar

**DOI:** 10.3390/plants12030475

**Published:** 2023-01-19

**Authors:** Finiavana Mihary Valisoa Rakotonirina, Dario Donno, Zoarilala Rinah Razafindrakoto, Nantenaina Tombozara, Roger Marie Rafanomezantsoa, Charles Andrianjara, David Ramanitrahasimbola, Gabriele Loris Beccaro

**Affiliations:** 1 Centre Hospitalier Universitaire Andrainjato Fianarantsoa, Faculté de Médecine, Université de Fianarantsoa, Antananarivo 101, Madagascar; 2 Ecole Doctorale de Geochimie et Chimie Médicinale, Université de Fianarantsoa, Antananarivo 101, Madagascar; 3 Dipartimento di Scienze Agrarie, Forestali e Alimentari, Università degli Studi di Torino, 10095 Grugliasco, Italy; 4 Institut Malgache de Recherches Appliquées, Antananarivo 101, Madagascar; 5 Mention Pharmacie, Faculté de Médecine, Université d’Antananarivo, Antananarivo 101, Madagascar

**Keywords:** tapia, phytochemicals, antioxidant capacity, analgesic activity, anti-inflammatory activity, antidiabetic activity, endemism, Madagascar

## Abstract

Antioxidants are important supplements for the human body for their roles in human life for the maintenance of homeostasis. Tapia fruits (*Uapaca bojeri*) are used by the riverain population of the Tapia forests in Madagascar as complementary foods. This study aims to quantify the main antioxidants in the *U. bojeri* fruits to verify their contribution to the enhancement of their anti-inflammatory and antihyperglycemic effects. Standard phytochemical screening was used for qualitative analysis, while spectrophotometric (TPC, TAC, and TFC) and chromatographic analyses (HPLC) were used to quantify several phytochemicals in *U. bojeri* fruits. The antioxidant activity was evaluated using DPPH and FRAP assays. The writhing test was used for the analgesic effects, the carrageenan-induced paw edema was used for the anti-inflammatory activity, and OGTT was used to test the anti-hyperglycemia property of the MEUB in mice. Several phytocompounds were detected and quantified in the fruits, including succinic acid (67.73%) as the main quantified compound. Fruits exerted a good antioxidant capacity and showed analgesic, anti-inflammatory, and antihyperglycemic activities in mice. Isolation of the bioactive compounds should be carried out to confirm these pharmacological properties and develop health-promoting food products or medicinal applications derived from this species.

## 1. Introduction

Antioxidant compounds play important roles in human life for the maintenance of homeostasis. The ingestion of exogenous antioxidants could help our organism to stabilize reactive oxygen species (ROS), which are harmful to cells and tissues [1,2]. An unbalance between ROS and natural antioxidants is the main cause of oxidative stress; in this case, ROS, mainly produced by the mitochondria in cells, cause several problems to other cells and tissues [3,4]. ROS are often composed of free radical atoms, molecules, or ions with unpaired electrons that are extremely unstable and susceptible to active chemical reactions with other molecules. Oxidative stress impairs several endothelial functions, vascular smooth muscle, and adventitial cells involved in several cardiovascular disorders, including hypertension, atherosclerosis, hypercholesterolemia, and diabetes (Cai and Harrison, 2000; Harrison et al., 2003) [5,6]. Moreover, during their respiration, leukocytes and activated macrophages produce free radicals, which damage epithelial and stromal cells leading to carcinogenesis by modifying targets and pathways essential for normal tissue homeostasis (Hussain et al., 2003) [7]. Antioxidant supplements are necessary in the case of pathologic excessive production of ROS. These compounds are often molecules possessing labile hydrogen that may interact with the ROS and produce stable radicals (e.g., phenolic and organic acid compounds). These compounds can be extracted from edible plants, including fruits and vegetables used in diets [8,9,10]. Moreover, alternative treatment with fruits showed a growing interest in a broad spectrum of diseases, including diabetes, obesity, neurodegenerative, gastric, inflammation and cardiovascular disorders, and certain types of cancers [11,12,13,14,15].

**Figure 1:**
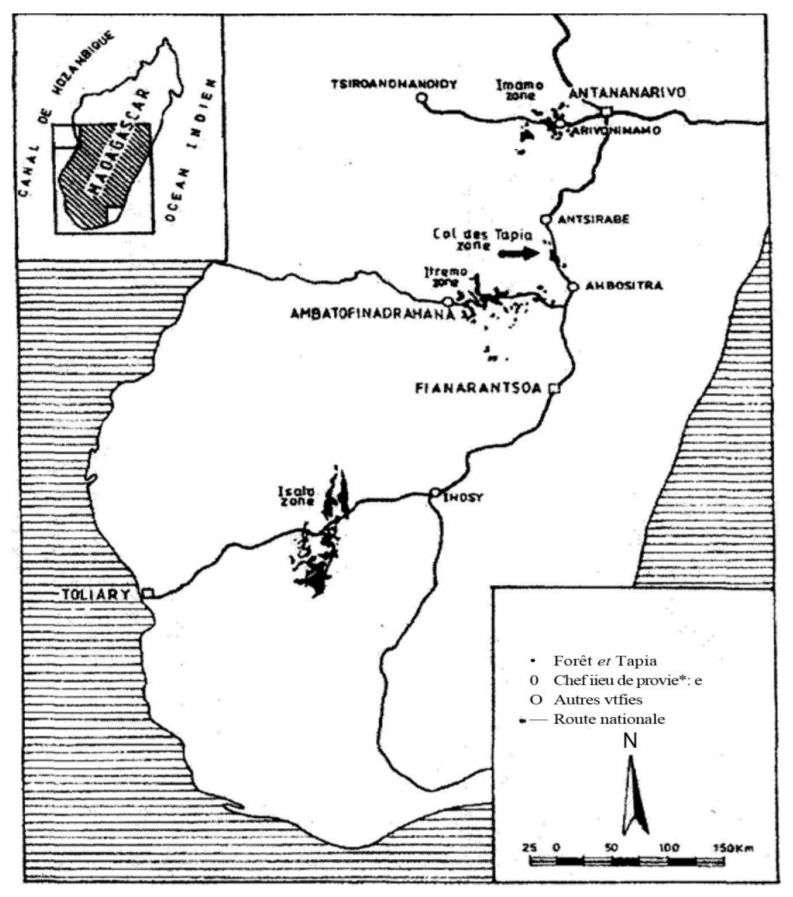
Tapia forests distribution in Madagascar.

Tapia, also known by its botanical name *Uapaca bojeri* Bail. (*Euphorbiaceae*), is an endemic tree species from the Tapia forests of Madagascar (Figure 1) localized in the Imamo massif, near Arivonimamo and Miarinarivo, the Hill of Tapia in the region of Manandona between Antsirabe and Ambositra, the Itremo massif in the west of Ambatofinandrahana, and the Isalo massif near Ranohira [16]. The Tapia fruits have a drupe shape with a fleshy, sweet, sticky mesocarp; woody endocarp protecting the three seeds at maturity. The Tapia produces large quantities of small, juicy, oval, and edible fruits, called Voapaka or Voatapia in the Malagasy language. Its flowering period is between March and September, while its fruiting period is from mid-September to early December [17]. This species is known as a medicinal plant and possesses several health-promoting properties. The local population uses this plant in the treatment of diabetes, infectious diseases, and hypertension [18], and the rape fruits are used as a complementary food and source of income for the riverain population during the fructification period. Recently, several compounds have been quantified from the *U. bojeri* leaves and the stems, including carotenoids, organic acids, and phenolics which contribute to the antioxidant, antidiabetic, and anti-inflammatory properties of this species [19]. Leaves, stems, and fruits are the main storage of secondary metabolites in plants. Nowadays, the trend in the research of natural treatments is growing due to the side effects caused by long-term treatment with synthetic drugs [20,21,22,23]. As discussed in the study of Razafindrakoto et al. [19], the quantified antioxidant secondary metabolites in the methanol extracts of the *U. bojeri* leaves and stems contribute to the antidiabetic, antalgic, and anti-inflammatory activities of this species. However, few studies are reported in the literature on the phytochemical and biological characterization of *U. bojeri* fruits. For this reason, the main aim of this study was to identify and quantify the main bioactive compounds susceptible as antioxidants in the methanol extract of the *U. bojeri* fruits to verify their contribution to the enhancement of their potential therapeutic effects toward inflammatory diseases and diabetes. 

## 2. Results and Discussion

### 2.1. Phytochemicals and Antioxidant Properties

The methanol maceration produced an extract of *U. bojeri* fruits (MEUB) with a yield of 12.45% relative to the fresh powder. Classes of phytochemicals detected in the fruits of *U. bojeri* were reported in Table 1. 

Most of the main antioxidant phytochemicals, including flavonoids, anthocyanins, phenolics, and tannins, were identified in the fruits. These phytocompounds were quantitatively determined as total flavonoid content (TFC), total phenolic content (TPC), and total anthocyanin content (TAC). They showed values of 118.05 ± 31.05 mg_QE_/100 g fresh weight (FW), 883.60 ± 186.62 mg_GAE_/100 g FW, and 423.51 ± 14.89 mg_C3GE_/100 g FW (Table 2), respectively.

The amounts of the main bioactive compounds are reported in Table 3. Among the 28 biomarkers considered in this study, 14 molecules were detected and quantified; succinic acid was the main compound, with a percentage of 67.73% in relation to the total bioactive compound content (TBCC) that was represented by the sum of all the quantified compounds. Organic acids were the main class of detected phytocompounds, with a percentage of 79.56% in relation to TBCC, in particular succinic, quinic, and oxalic acids. Succinic acid was also the main compound quantified in leaves and the stems with values of 533.74 ± 340.08 and 1275.65 ± 434.99 mg/100 g dry weight, respectively [19], showing that this plant may be considered a good source of succinic acid for the growing interest in pharmaceutical, agricultural, food, and chemical industry applications [24]. Cinnamic acids were represented by caffeic and chlorogenic acids (0.04% and 1.09%, respectively); moreover, four flavonols were detected, including hyperoside (0.03%), isoquercitrin (0.05%), quercetin (0.73%), and quercitrin (3.57%). Ellagic acid (8.34%) represented the class of benzoic acids, and epicatechin (0.84%) represented the class of catechins, while castalagin (4.20%) and vescalgin (0.94%) were detected in the class of tannins. Vitamin C (0.60%) was also identified and quantified in the samples. Most of these quantified compounds show labile hydrogens and could stabilize ROS.

The antioxidant capacity of the *U. bojeri* fruits was evaluated using the free radical DPPH assay on the MEUB and confirmed with the FRAP assay. The DPPH assay is based on the ability of the compounds in the extracts to donate hydrogen to the radical DPPH (used as the ROS model) and form stable DPPH-H and stable radicals, while the FRAP assay is based on the capacity of the compounds in the plants to give electrons to the ferric ions and form ferrous ions and stable radicals [25]. The results are reported in Table 2. The fruit extracts averagely inhibited the radical DPPH with an IC_50_ of 301.85 ± 3.62 µg/mL in relation to the gallic acid (*p* < 0.05), higher values than leaf (47.36 ± 3.00 µg/mL; *p* < 0.05) and stem (33.32 ± 0.69 µg/mL; *p* < 0.05) extracts [19]. 

The FRAP results showed that the fruits were two-fold less active than the leaves and stems. Effectively, its capacity to reduce Fe^3+^-TPTZ into Fe^2+^-TPTZ is two-fold less important (39.72 ± 0.34 mmol Fe^2+^/kg FW) than that of leaves (69.20 ± 1.41 mmol DW [19]) or stems (70.17 ± 9.53 mmol Fe^2+^/kg DW [19]). It may be due to the TPC content. Indeed, fruits contain less TPC than leaves (3624.72 ± 268.07 mg_GAE_/100 g FW) or stems (5854.17 ± 1247.67 mg_GAE_/100 g FW). Moreover, several studies showed the relationship between the phenolics, organic acids, flavonoids, and vitamin content and the antioxidant capacity that could influence the AOC values in several plant materials [16,19,26,27]. 

### 2.2. Analgesic Effect of MEUB

The analgesic activity of MEUB was evaluated using the writhing method, one of the most common peripheral analgesic animal models for the screening of analgesic drugs [28]. The number of writhing and the percentage of inhibition exerted by the animals after the treatment with MEUB were reported in Table 4. The intraperitoneal injection of 1% acetic acid solution into the control group caused 24.6 ± 4.3 writhing between the intervals of 25 min after the 5th min of acetic acid induction. The treatment with MEUB at each dose significantly decreased the number of writhing (*p* < 0.01) in a dose-dependent manner (*p* < 0.001 with ANOVA). The paracetamol at the dose of 100 mg/kg inhibited the pain caused by the acetic acid induction at 82.11%, showing the effectiveness of the protocol. MEUB at the dose of 200 and 400 mg/kg exerted similar analgesic activity when compared to the paracetamol 100 mg/kg (*p* ˃ 0.05). The peripheral analgesic effect may be mediated by the inhibition of cyclo-oxygenases and/or lipoxygenases (and other inflammatory mediators), while the central analgesic action may be mediated by the inhibition of central pain receptors. Several classes of phytocompounds identified in this species exerted these properties (e.g., steroids, flavonoids such as quercetin and quercitrin, and tannins such as castalagin and vescalgin) [29,30,31,32,33], showing their contribution to the pain reduction effect of the MEUB. The pain inhibition effect of the ripe fruit is better than the effect of leaves (49.40% vs. 70.73% at the dose of 400 mg/kg) and stems (57.43 vs. 70.73% at the dose of 400 mg/kg), showing the importance of the other compounds detected in the fruits (e.g., steroids) [19].

### 2.3. Anti-Inflammatory Effect of MEUB

The anti-inflammatory effect of the MEUB was evaluated using carrageenan-induced mice paw edema, one of the most used in vivo protocols for the evaluation of the anti-inflammatory activity of natural products [33]. The injection of carrageenan (2%) caused inflammation at the mice’s paw level that was progressively reduced after the treatment. The inflammatory response was detected in two phases; the first phase occurred between the 0 and 120th min post carrageenan injection due to the release of histamine, serotonin, and bradykinin. These mediators increased vascular permeability in the surrounding damaged tissues [34]. The second phase occurred after the 120th min and was identified together with the biosynthesis of prostaglandins and infiltration of neutrophils [35]. The treatment with MEUB at the dose of 100, 200, and 400 mg/kg significantly reduced the inflammation at 60, 120, 180, and 240 min (*p* < 0.05, *p* < 0.01, and *p* < 0.001; Table 5) compared with the negative control. The effect of MEUB was in a dose-dependent manner after 180 and 240 min (*p* < 0.001, respectively). The effect of the MEUB was comparable to the effect of the indomethacin at the dose of 10 mg/kg at the 120th, 180th, and 240th min post carrageenan injection (*p* > 0.05), showing that the MEUB was more effective during the second phase (however, the inhibition of the first phase should not be unnoticed during the release of prostaglandins). These observations showed that the secondary metabolites in the MEUB could antagonize the release and/or the activity of the chemical mediators during both phases. Compared to the leaf and stem extracts, as reported by Razafindrakoto et al. [19], the MEUB was more effective at higher doses (200 and 400 mg/kg) after the 60th, 120th, and 180th min carrageenan injection. It may be due to the high rate of succinic acid in the fruit, with a value of 67.73% vs. 32.68% in the leaves and 41.64% in the stems. Succinic acid and derivatives are reported as a potent anti-inflammatory by increasing TNF-α secretion and suppressing IL-6 production in LPS-stimulated cells [36]. Moreover, Chang et al. [37] reported the contribution of vescalagin as an antioxidant in its anti-inflammatory properties; on the other hand, Al-Sayed and Abdel-Daim [31] also demonstrated the antioxidant and anti-inflammatory activity of epicatechin.

### 2.4. Antihyperglycemic Activity

The effects of MEUB on the blood glucose level were evaluated using an oral glucose tolerance test (OGTT) in mice. The results are reported in Table 6. As shown in Table 6, the glycemia increased from the administration of glucose (t = 0 min) to the 30th min, then it gradually decreased from the 60th min for each treatment showing that the body releases insulin to regulate the rate of glucose and keep the homeostatic state and demonstrating the effectiveness of the protocol. The glycemia dose-dependently decreased after the 60th, 90th, 120th, and 150th min (*p* < 0.01) glucose loading, and already after 30 min from the glucose administration, mice fed with MEUB at 200 and 400 mg/kg doses already showed lower glucose values compared to those of negative control. Compared to the GBC, after the 60th min glucose loading, MEUB at the dose of 200 mg/kg showed a silent, low effect in terms of variation of glycemia, but the glycemia values did not show any statistical difference compared to GBC; moreover, at the dose of 400 mg/kg, the glycemia variation was higher for MEUB than glycemia variation of GBC, but the glucose level did show any statistical difference. Benzoic acid-related molecules, castalagin, vescalagin, and other antioxidant compounds enhanced insulin effects and reduced insulin resistance [37,38] and the improvement of liver; cardiovascular and metabolic parameters in a rat model of human diseases [39] could be at the origin of these effects on the glycemia decreasing. Additionally, epicatechin, a potent antioxidant that lowers blood pressure [40], plays an important role as insulin-like, contributing to the antihyperglycemic activity of the *U. bojeri* fruits [41].

## 3. Materials and Methods

### 3.1. Plant Materials

The ripe fruits of *U. bojeri* were collected in November 2019 in the Imamo forests in the district of Miarinarivo, about 70 km from Antananarivo. A specimen was identified by Mr. Benja Rakotonirina, the botanist of the *Institut Malgache de Recherches Appliquées* (IMRA), and the voucher specimen was compared to the previous reference TN-021/LPA at the IMRA Botanical Department. The ripe fruits were stored in a cool and airy place away from sunlight before being ground.

### 3.2. Animals

Swiss albino male and female mice (weight: 25 ± 5 g; age: 4–5 months), kept under controlled conditions (12 h dark and 12 h light cycle, 25 ± 2 °C temperature, and 50 ± 10% humidity) at the IMRA animal house, were used. The animals received standard food pellets (1420, Livestock Feed Ltd., Port Louis, Mauritius), and they remained fasting for one night before the experiment. All experiments were carried out following the DIRECTIVE 2010/63/EU and approved by the local ethic committee (number: 06/CEA-IMRA/2020).

### 3.3. Chemicals and Reagents

The chemicals and reagents used during the study are reported in Supplementary Material. 

### 3.4. Qualitative Analysis

The classes of secondary metabolites were detected by the traditional methods for phytochemical screening described in the work of Tombozara et al. [42]. Their principle is based on the formation of colored soluble or precipitated compounds by the specific reactive reagent used. 

### 3.5. Quantitative Analysis

#### 3.5.1. Total Phenolic Content (TPC), Total Anthocyanin Content (TAC), and Total Flavonoid Content (TFC)

The protocol described by Slinkard and Singleton [43] was used to evaluate the TPC in triplicate using the Folin–Ciocalteu reagent. TAC was determined using the pH differential method in triplicate described in the protocol of Lee et al. [44]. The method is based on the coloration change of the colored monomeric anthocyanin in oxonium form when diluted at pH 1.0 to the colorless hemiketal form at pH 4.5. The aluminum chloride (AlCl_3_) method was used for the determination of the TFC in triplicate, according to Matic et al. [45]. The details are reported in Supplementary Material.

#### 3.5.2. HPLC Analysis

The samples for the HPLC analysis of phytoconstituents were prepared in triplicate according to the method described by Razafindrakoto et al. [19]. The extraction protocol was reported in Supplementary Material. Twenty-eight standards (Table 3) were selected and manually injected (20 µL) in triplicate for their quantification in this study. The quantitation of these compounds was performed using an Agilent 1200 High-Performance Liquid Chromatograph coupled to an Agilent UV-Vis diode array detector (Agilent Technologies, Santa Clara, CA, USA) according to the protocol described by Razafindrakoto et al. [19]. 

### 3.6. Antioxidant Activity Evaluation

The antioxidant activity of the methanolic extract of *U. bojeri* (MEUB) was determined using the free radical DPPH assay along with the FRAP assay that is based on the capacity of the sample to reduce the ferric ions Fe^3+^ into ferrous ions Fe^2+^ in the 2,4,6-tripyridyl-s-triazine (TPTZ) complex [46]. Both protocols have been described by Razafindrakoto et al. [19], and they are detailed in Supplementary Material.

### 3.7. Acetic-Acid-Induced Writhing Test

The protocol of Olajide et al. [47], slightly modified by Razafindrakoto et al. [19], was used to determine the analgesic property of MEUB (Supplementary Material).

### 3.8. Carrageenan-Induced in Paw Oedema Test

In vivo anti-inflammatory activity was evaluated based on the inhibition of carrageenan-induced mouse hind paw edema using a plethysmometer as previously described by Buisseret et al. [48], slightly modified by Razafindrakoto et al. [49] (Supplementary Material).

### 3.9. Oral Glucose Tolerance Test (OGTT)

OGTT, described by Tombozara et al. [16] with slight modifications, was applied to determine the hypoglycemia property of MEUB (Supplementary Material).

### 3.10. Statistical Analysis

The results were expressed as mean ± standard error means (S.E.M.), and the data were statistically analyzed using the Student’s *t*-test and one-way analysis of variance (ANOVA) followed by the HSD Tukey multiple range test using SPSS 20.0 software. All the differences showing a *p* < 0.05 were accepted as statistically significant.

## 4. Conclusions

This work was performed to determine the antioxidant compounds in the fruit of *U. bojeri*, consumed by the local people living around the Tapia forests, and their contribution to the management of metabolic diseases, including inflammatory and related diseases and diabetes. Overall, 14 compounds belonging to phenolic, flavonoid, vitamin, and organic acid classes have been quantified in the fruit of *U. bojeri,* contributing to its antioxidant, analgesic, anti-inflammatory, and antihyperglycemic activities. The tested product is a methanolic crude extract (MEIC) of the *U. bojeri* fruit, which is a mixture of more secondary metabolites. Some of them could contribute to the pharmacological activity. In the case of the mixture of active compounds, the synergy of action can be envisaged, but all molecules have their pharmacokinetic profile. It is therefore not entirely obvious to have a dose-dependent response as in the case of an isolated bioactive molecule. This study contributed to the valorization of this species as a health promotor, mostly as complementary medicine in the treatment of diabetes and inflammatory and related diseases, but further studies, including the isolation of the bioactive compounds, should be performed to confirm the properties of this plant. It is very important to investigate the local use of biodiversity to identify new bioactive compounds for supporting biodiversity conservation and sustainable development projects in Madagascar.

## Figures and Tables

**Table 1:** Classes of secondary metabolites in the fruit of *U. bojeri*.

Class of Secondary Metabolites	*U. Bojeri* Fruits
Flavonoids	+
Anthocyanins	+
Leucoanthocyanins	+
Phenolic compounds	+
Tannins	+
Terpenoids	−
Alkaloids	−
Steroids	+
Lactonic steroids	−
Unsaturated sterols	+
Cardiac glycosides	−
Quinones	
Polysaccharides	−
Saponins	+

+ as the presence of phytochemicals; − as the absence of phytochemicals.

**Table 2:** TPC, TFC, TFC, and FRAP result from the fruit of *U. bojeri* and the IC_50_ of MEUB on the radical DPPH.

Assay/Sample	Fruit Extract	Gallic Acid
TPC (mgGAE/100 g FW)	883.60 ± 186.62	-
TAC (mgC3GE/100 g FW)	423.51 ± 14.89	-
TFC (mgQE/100 g FW)	118.05 ± 31.05	-
FRAP (mmol Fe^2+^/kg of FW)	39.72 ± 0.34	-
DPPH: IC_50_ (µg/mL)	301.85 ± 3.62 ^a^	20.73 ± 1.35

Values are expressed as mean ± SD (*n* = 3); a: *p* < 0.05 vs. gallic acid (student *t*-test); GAE: gallic acid equivalent; C3GE: cyanidin-3-glycoside equivalent; QE: quercetin equivalent.

**Table 3:** Phytochemical contents in the fruit of *U. bojeri*.

Class	Standards	Rt (min)	λ (nm)	Value (mg/100 g FW)	Rate ^a^ (%)
Cinnamic acids	Caffeic acid	4.54	330	0.71 ± 0.13	0.04
	Chlorogenic acid	3.89	330	19.18 ± 0.38	1.09
	Coumaric acid	6.74	330	ND	0
	Ferulic acid	7.99	330	ND	0
Flavonols	Hyperoside	10.89	330	0.59 ± 0.23	0.03
	Isoquercitrin	11.24	330	0.90 ± 0.06	0.05
	Quercetin	17.67	330	13.08 ± 2.62	0.74
	Quercitrin	13.28	330	62.96 ± 18.78	3.57
	Rutin	12.95	330	ND	0
Benzoic acids	Ellagic acid	18.65	280	147.14 ± 6.53	8.34
	Gallic acid	4.26	280	ND	0
Catechins	Catechin	10.31	280	ND	0
	Epicatechin	14.30	280	14.86 ± 2.91	0.84
Tannins	Castalagin	16.35	280	74.02 ± 4.21	4.20
	Vescalagin	17.25	280	16.65 ± 7.62	0.94
Vitamins	Ascorbic acid	4.14	261	ND	0
	Dehydroascorbic acid	3.41	348	10.50 ± 3.22	0.60
Organic acids	Citric acid	5.30	214	ND	0
	Malic acid	4.04	214	ND	0
	Oxalic acid	7.85	214	70.49 ± 17.17	4.00
	Quinic acid	3.21	214	138.03 ± 23.02	7.83
	Succinic acid	3.46	214	1194.38 ± 269.07	67.73
	Tartaric acid	5.69	214	ND	0
Monoterpenes	Limonene	3.35	250	ND	0
	Phellandrene	3.57	210	ND	0
	Sabinene	3.45	220	ND	0
	γ-terpinene	3.28	235	ND	0
	Terpinolene	4.83	220	ND	0
TBCC				1763.50 ± 355.95	100

Values are expressed as mean ± SD of the amount of phytochemicals (*n* = 3). Rt: Retention time; λ: Wavelength; ND: Not detected; a: Relative to TBCC (Total bioactive compound content).

**Table 4:** Effects of MEUB on acetic acid-induced mice by *i.p*. route (*n* = 5).

Sample	Dose (mg/kg)	Writhing Number	Inhibition (%)
Vehicle	-	24.6 ± 4.3	-
Paracetamol	100	4.4 ± 0.7 ^a^	82.11
MEUB	100	8.6 ± 1.3 ^a,b^	65.04
	200	7.4 ± 1.2 ^a^	69.92
	400	7.0 ± 0.9 ^a^	71.54

Writhing numbers were expressed as mean ± SEM; a: *p* < 0.01 vs. vehicle; b: *p* ˂ 0.05 vs. paracetamol. Student *t*-test and ANOVA, followed by Tukey posthoc test, were performed.

**Table 5:** Effects of MEUB on carrageenan-induced mice hind paw edema (*n* = 5).

Sample	Dose (mg/kg)	Inhibition of Inflammation (%)				
		30 min	60 min	120 min	180 min	240 min
Vehicle	-	4.17 ± 2.04	11.50 ± 2.05	15.39 ± 2.88	20.32 ± 3.96	27.77 ± 4.35
Indomethacin	10	34.78 ± 6.00 ^b^	59.98 ± 6.00 ^a^	74.63 ± 5.83 ^a^	88.69 ± 5.02 ^a^	91.80 ± 2.02 ^a^
MEUB	100	15.90 ± 6.89	36.83 ± 4.54 ^b^	44.33 ± 6.01 ^b^	51.60 ± 5.41 ^b^	72.81 ± 8.13 ^b^
	200	10.87 ± 5.11	30.31 ± 9.09	67.00 ± 7.55 ^a^	81.77 ± 3.25 ^a^	91.08 ± 2.98 ^a^
	400	15.88 ± 5.81	36.04 ± 6.96 ^c^	62.85 ± 11.02 ^b^	91.40 ± 8.05 ^a^	93.99 ± 5.46 ^a^

a: *p* < 0.001; b: *p* < 0.01; c: *p* < 0.05 vs. vehicle. Student *t*-test and ANOVA, followed by Tukey posthoc test, were performed.

**Table 6:** Effect of MEUB and GBC on blood glucose level in mice (mg/dL).

Sample (*n* = 5)	Dose (mg/kg)	Time (min)						
		−60	0	30	60	90	120	150
Negative control	0	102.20 ± 2.37	99.80 ± 2.23	316.80 ± 37.32 0%	285.20 ± 37.04 14.56%	204.20 ± 28.09 51.89%	134.40 ± 6.03 84.06%	122.60 ± 6.24 89.49%
MEUB	100	101.60 ± 5.64	93.50 ± 5.52	333.20 ± 26.35 0%	282.20 ± 29.60 ^d^ 21.27%	202.20 ± 17.43 ^d^ 54.65%	141.60 ± 7.02 ^d^ 79.93%	122.60 ± 5.30 ^d^ 87.86%
	200	105.00 ± 4.27	101.25 ± 4.17	287.80 ± 22.63 0%	190.80 ± 16.23 ^b^ 52.00%	133.80 ± 5.02 ^c,d^ 82.55%	105.80 ± 4.37 ^b^ 97.56%	97.60 ± 7.07 ^c^ 101.96%
	400	90.60 ± 7.17	93.75 ± 6.68	274.40 ± 10.81 0%	143.60 ± 15.55 ^b^ 72.41%	104.40 ± 4.93 ^b^ 94.10%	95.60 ± 4.46 ^b^ 98.98%	89.80 ± 8.33 ^c^ 102.19%
GBC	10	106.00 ± 3.71	103.00 ± 3.52	302.00 ± 20.38 0%	171.20 ± 7.30 ^c^ 65.73%	101.60 ± 6.50 ^b^ 100.70%	91.40 ± 5.25 ^a^ 105.83%	85.20 ± 5.58 ^b^ 108.94%

The above values are expressed as mean ± SEM of glycemia (*n* = 5). Below values are the percentage of glycemia variation relative to the glycemia variation at the 30th min. MEUB: Methanol extract of *U. bojeri*; GBC: Glibenclamide a: *p* ˂ 0.001 vs. negative control; b: *p* ˂ 0.01 vs. negative control; c: *p* ˂ 0.05 vs. negative control; d: *p* ˂ 0.05 vs. GBC. Student *t*-test and ANOVA, followed by Tukey posthoc test, were performed.

## Data Availability

Not applicable.

## Supplementary Materials

The following supporting information can be downloaded at: https://www.mdpi.com/article/10.3390/plants12030475/s1.
