# Supplementary material for: Quali–Quantitative Fingerprinting of the Fruit Extract of Uapaca bojeri Bail. (Euphorbiaceae) and Its Antioxidant, Analgesic, Anti-Inflammatory, and Antihyperglycemic Effects: An Example of Biodiversity Conservation and Sustainable Use of Natural Resources in Madagascar
Source: Plants (Basel). 2023 Jan 19;12(3):475. doi: 10.3390/plants12030475 (PMC9921778; doi:10.3390/plants12030475)
Supplement: Supplementary file 1 [file plants-12-00475-s001.zip › plants-2086800-supplementary.pdf]

plants

MDPI

Article

Quali–Quantitative Fingerprinting of the Fruit Extract of *Uapaca bojeri* Bail. (*Euphorbiaceae*) and Its Antioxidant, Analgesic, Anti-Inflammatory, and Antihyperglycemic Effects: An Example of Biodiversity Conservation and Sustainable Use of Natural Resources in Madagascar

Finiavana Mihary Valisoa Rakotonirina [1,2], Dario Donno [3,*], Zoarilala Rinah Razafindrakoto [4], Nantenaina Tombozara [4], Roger Marie Rafanomezantsoa [2], Charles Andrianjara [4], David Ramanitrahasimbola [4,5] and Gabriele Loris Beccaro [3]

1 Centre Hospitalier Universitaire Andrainjato Fianarantsoa, Faculté de Médecine, Université de Fianarantsoa, Antananarivo 101, Madagascar
2 Ecole Doctorale de Geochimie et Chimie Médicinale, Université de Fianarantsoa, Antananarivo 101, Madagascar
3 Dipartimento di Scienze Agrarie, Forestali e Alimentari, Università degli Studi di Torino, 10095 Grugliasco, Italy
4 Institut Malgache de Recherches Appliquées, Antananarivo 101, Madagascar
5 Mention Pharmacie, Faculté de Médecine, Université d'Antananarivo, Antananarivo 101, Madagascar
* Correspondence: dario.donno@unit.it; Tel.: +39-0116-708751

1. Methods

1.1. Chemicals and reagents

Sodium carbonate, Folin–Ciocalteu phenol reagent, sodium acetate, acetic acid, citric acid, aluminium chloride, sodium nitrite, potassium chloride, hydrochloric acid, iron (III) chloride hexahydrate, 2,4,6-tripyridyl-S-triazine, 1,2-phenylenediamine dihydrochloride (OPDA), all polyphenolic and terpenic standards, potassium dihydrogen phosphate, phosphoric acid, glibenclamide, 1,1-diphenyl-2-picrylhydrazyl (DPPH), carrageenan, indomethacin, and HPLC-grade methanol and acetonitrile were purchased from Sigma-Aldrich (St. Louis, MO, USA). Acetic acid, ethanol, organic acids, and HPLC-grade formic acid were purchased from Fluka BioChemika, Buchs, Switzerland. Ethylenediaminetetraacetic acid disodium salt was purchased from AMRESCO (Solon, OH, USA). Sodium fluoride was purchased from Riedel-de Haen (Seelze, Germany). Cetyltrimethylammonium bromide (cetrimide), ascorbic acid (AA), and dehydroascorbic acid (DHAA) were purchased from Extrasynthèse (Genay, France). Milli-Q ultrapure water was produced by Sartorius Stedim Biotech mod. Arium (Sartorius, Göettingen, Germany).

1.2. Qualitative analysis

The classes of secondary metabolites were detected by the classical methods for phytochemical screening described in the work of Tombozara *et al.* (2017). Their principle is based on the formation of coloured soluble or precipitated compounds by the specific reactive reagent used.

1.3. Quantitative analysis

1.3.1. Total phenolic content (TPC)

The protocol described by Slinkard and Singleton (1977) was used to evaluate the TPC in triplicate using the Folin–Ciocalteu reagent. Briefly, 5 g of the rape fruit powder were macerated in 35 mL of a mixture of methanol-water (95:5 v/v) solution acidified by

hydrochloric acid in the dark for 24 h. The mixture was filtered and 200 μL of the filtrate was added to 1 mL of the Folin–Ciocalteu reagent (dilution 1:10) and 800 μL of sodium carbonate (7.5%). The mixture was stored in the dark for 30 min and the absorbance was measured at 765 nm. The results were expressed as mg gallic acid equivalents (GAE)/100 mg of fresh weight (FW) where the gallic acid was used as the standard solution and prepared at a different concentration varying from 0.02 to 0.10 mg/mL.

1.3.2. Total anthocyanin content (TAC)

TAC was determined using the pH differential method in triplicate described in the protocol of Lee *et al.* (2005). The method is based on the change in colouration of the coloured monomeric anthocyanin in oxonium form when diluted at pH 1.0 to the colourless hemiketal form at pH 4.5. Potassium chloride buffer (0.025 M) was prepared, and the pH was adjusted to 1.0 with HCl and acetate buffer (sodium acetate 0.4 M) was prepared and the pH was adjusted to 4.5 with HCl. The sample was prepared the same as in the TPC determination method and diluted in both prepared buffer solutions. The absorbance was measured at both 520 and 700 nm using a spectrophotometer (Genesis) and the results were expressed as cyanidin-3-glucoside equivalents (C3GE) in mg/100 g FW.

1.3.3. Total flavonoid content (TFC)

The aluminium chloride ($AlCl_3$) method was used for the determination of the TFC in triplicate according to Matic *et al.* (2017). The sample was prepared the as in the TPC determination method slightly modified. Sample (200 μL) along with 800 μL of methanol-water (95/5; v/v) was added to 60 μL of sodium nitrite (5%) in a spectrophotometric cuvette. After 5 min incubation, 60 μL of $AlCl_3$ (10% in methanol), 400 μL NaOH (1 mol/L) and 480 μL of methanol (95%) were added to the cuvette. The mixture was vortexed, and the absorbance was measured with a spectrophotometer (Genesis) at 510 nm. Methanol (95%) was used as blank and quercetin with a range concentration of 1 – 500 μg/mL was used as the standard for the calibration curve determination. The results were expressed as quercetin equivalent (QE) in mg/100 g FW.

1.3.4. HPLC analysis

The samples for the HPLC analysis of phytoconstituents were prepared in triplicate according to the method described by Razafindrakoto *et al.* (2020). Fresh plant powder (5 g) was macerated in 35 mL of methanol-water (95/5;v/v) acidified with hydrochloric acid for 24 h in dark then mixed and homogenised with a mixer for 3 min before incubation in dark for 24 h. The mixture was filtered, and the filtrate was stored at 4°C. A second maceration with the same solvent was performed with the marc for 72 h in dark. After filtration, both filtrates were gathered and stored under normal conditions before use.

The quantitation of bioactive compounds was performed using an Agilent 1200 High-Performance Liquid Chromatograph coupled to an Agilent UV-Vis diode array detector (Agilent Technologies, Santa Clara, CA, USA) according to the protocol described by Razafindrakoto *et al.* (2020). The separation was done using five chromatographic methods (A-E) on a Kinetex C18 column (4.6 × 150 mm, 5 μm, Phenomenex, Torrance, CA, USA) and different mobile phases, where the UV spectra and the retention times were recorded at different wavelengths for the identification of each biomarker.

1.4. Preparation of the methanol extract of U. bojeri (MEUB)

Fresh ground plants (100 g) were macerated with 300 mL in a mixture of methanol-water (95-05, v-v) for 24 h before filtration. The plant material was again extracted with the same solvent twice to get an efficient extraction following the same process. The filtrates were gathered and evaporated under reduced pressure at 40°C to obtain the methanol extract which was stored at 4 °C until analysis.

1.5. Antioxidant activity evaluation

1.5.1. Free radical DPPH scavenging capacity

The free radical DPPH assay described in the study of Razafindrakoto *et al.* (2020) was used to evaluate the capacity of MEUB to inhibit free radicals. MEUB methanolic solution (25 µL) at different concentrations (7.8, 15.6, 31.25, 62.5, and 125 µg/mL) was added to 175 µL of methanol DPPH solution (0.25 mmol/L) in a 96-well microplate and incubated at room temperature for 30 min. Methanol was used as a blank and a methanol solution of DPPH was used as a negative control. Gallic acid (2.5, 5, 10, 20, and 40 µg/mL) was used as positive control and the results were expressed as inhibiting concentration (IC), calculated using the following equation: IC (%) = 100 × (A_0 – A_1) / A_0, where A_0 and A_1 are the values for the absorbance of the negative control and sample, respectively, measured at 517 nm with a spectrophotometer. The IC_{50} (inhibition concentration at 50%) values of MEUB and gallic acid were calculated by linear regression.

1.5.2. Ferric reducing antioxidant power (FRAP)

The FRAP assay is based on the capacity of the sample to reduce the ferric ions Fe^{3+} into ferrous ions Fe^{2+} in the 2,4,6-tripyridyl-s-triazine (TPTZ) complex (Benzie and Strain, 1999). The protocol described by Razafindrakoto *et al.* (2020) was used. The sample was prepared the same as in the TPC determination. Then, 30 µL of the sample were mixed with 90 µL of distilled water and 900 µL of FRAP reagent composed of a mixture of TPTZ and $FeCl_3{\cdot}6H_2O$ solutions with acetate buffer (0.3 M). The mixture was stirred and incubated at 37 °C for 30 min. The absorbance was read at 595 nm using a UV-Vis spectrophotometer (UV–1600PC, VWR, Milan, Italy). The standard curve was obtained using $FeSO_4{\cdot}7H_2O$ with concentrations ranging from 100 to 1000 mmol/L, and the results were expressed as millimoles of ferrous ion equivalent per kilogram FW.

1.6. Acetic acid-induced writhing test

The protocol of Olajide et *al.*, (2000), slightly modified by Razafindrakoto *et al.* (2020) was used to determine the analgesic property of MEUB. Five groups of 5 fasted mice were used during the assays. Distilled water was orally administered to group I which was used as the negative control, while paracetamol at a dose of 100 mg/kg *b.w.* was administered, by gavage, to the positive control group (group II); MEUB at a dose of 100, 200 and 400 mg/kg *b.w.* was administered to group III – V respectively one hour before the injection of 100 µL of acetic acid solution (1%) in 0.9% saline solution by *i.p.* route to induce characteristic writhing. The number of writhing occurring between 5 and 30 min after acetic acid injection was counted.

1.7. Carrageenan-induced in paw oedema test

In vivo anti-inflammatory activity was evaluated based on the inhibition of carrageenan-induced mouse hind paw oedema using a plethysmometer as previously described by Buisseret et *al.* (2019), slightly modified by Razafindrakoto *et al.* (2021). Briefly, fasted mice were divided into 5 groups of 5 mice. Group I orally received distilled water and served as the negative control. Group II, orally indomethacin administered (10 mg/kg *per os*), was the positive control group and Groups III - V were fed by MEUB, respectively, at a dose of 100, 200 and 400 mg/kg (*b.w.*) one hour before the induction of 100 µl of carrageenan solution (2%) in normal saline solution (0.9%) into the right hind paw of each mouse. Paw volume was measured using a water plethysmometer (Ugo Basile 7140, Italy) before and 30, 60, 120, 180 and 240 min after the carrageenan injection. The percentage of the inhibition of the inflammation (II) was calculated using the following formulae: II (%) = ((V_0 – V_i) – (V_t – V_i)) x 100 / (V_0 – V_i) where V_0 and V_t were the paw volume 0 and t min after the carrageenan injection, respectively, and V_i was the paw volume before the carrageenan injection.

1.8. Oral glucose tolerance test (OGTT)

OGTT described by Tombozara *et al.* (2020) with slight modifications was applied to determine the hypoglycaemia property of MEUB. Fasted mice were divided into five groups of five animals. Group I received distilled water and served as negative control; group II was treated with glibenclamide (GBD) at 10 mg/kg body weight (b.w.), and group III – V received, respectively, 100, 200, and 400 mg/kg b.w. of MEUB 60 min before administration of 4 g/kg b.w. glucose (t = 0 min). Glucose level was measured from the venous blood sample taken at the end of the tail using a glucometer (Senso Card) at t = −60 min (before the treatment), t = 0 (before administration of glucose), t = 30, 60, 90, 120 min, and 150 min (after the glucose loading).
